# Spatial and Temporal Variation of Land Surface Temperature and Its Spatially Heterogeneous Response in the Urban Agglomeration on the Northern Slopes of the Tianshan Mountains, Northwest China

**DOI:** 10.3390/ijerph192013067

**Published:** 2022-10-11

**Authors:** Xueling Zhang, Alimujiang Kasimu, Hongwu Liang, Bohao Wei, Yimuranzi Aizizi

**Affiliations:** 1 School of Geography and Tourism, Xinjiang Normal University, Urumqi 830054, China; 2 Research Centre for Urban Development of Silk Road Economic Belt, Xinjiang Normal University, Urumqi 830054, China; 3 Xinjiang Key Laboratory of Lake Environment and Resources in Arid Zone, Urumqi 830054, China

**Keywords:** land surface temperature, arid zone urban agglomeration, trend change analysis, landscape pattern, GD, MGWR

## Abstract

An in-depth study of the influence mechanism of oasis land surface temperature (LST) in arid regions is essential to promote the stable development of the ecological environment in dry areas. Based on MODIS, MYD11A2 long time series data from 2003 to 2020, the Mann–Kendall nonparametric test, the Sen slope, combined with the Hurst index, were used to analyze and predict the trend of LST changes in the urban agglomeration on the northern slopes of the Tianshan Mountains. This paper selected nine influencing factors of the slope, aspect, air temperature, normalized vegetation index (NDVI), precipitation (P), nighttime light index (NTL), patch density (PD), mean patch area (AREA_MN), and aggregation index (AI) to analyze the spatial heterogeneity of LST from global and local perspectives using the geodetector (GD) model and multi-scale geo-weighted regression (MGWR) model. The results showed that the average LSTs of the urban agglomeration on the northern slopes of the Tianshan Mountains in spring, summer, autumn, and winter were 31.53 °C, 47.29 °C, 22.38 °C, and −5.20 °C in the four seasons from 2003 to 2020, respectively. Except for autumn, the LST of all seasons showed an increasing trend, bare soil and grass land had a warming effect, and agricultural land had a cooling effect. The results of factor detection showed that air temperature, P, and NDVI were the dominant factors affecting the spatial variation of LST. The interaction detection results showed that the interaction between air temperature and NDVI was the most significant, and the two-factor interaction was more effective than the single-factor effect on LST. The MGWR model results showed that the effects of PD on LST were positively correlated, and the impact of AREA_MN and AI on LST were negatively correlated, indicating that the dense landscape of patches has a cooling effect on LST. Overall, this study provides information for managers to carry out more targeted ecological stability regulations in arid zone oases and facilitates the development of regulatory measures to maintain the cold island effect and improve the environment.

## 1. Introduction

The land surface temperature (LST) is a mapping of the material exchange and energy flow between solar radiation, the atmospheric environment, and the Earth’s surface [1,2]. LST variations directly affect the magnitude of long-wave radiation energy in the ground air system and fluctuations in climate elements, such as near-surface air temperature, ground mass evapotranspiration, and atmospheric relative humidity [3]. Additionally, these effects are widely applied to fields such as agricultural production, water conservancy, hydropower projects, and ecological civilization construction [4]. Maintaining the appropriate temperature for the human environment and public health also provides security [5]. However, the thermal distribution at the surface is heterogeneous due to the different physical properties of the subsurface cover [6]. Therefore, today’s LST distribution is a combined product of natural and anthropogenic factors [7]. Accelerated urbanization has resulted in the encroachment of urban impervious surfaces on agricultural land and natural ecosystems, which significantly impacts urban energy consumption, thermal environmental characteristics, runoff magnitude, vegetation cover, and local climate [8]. The quantitative analysis of the spatial and temporal evolution of LST and its drivers at the urban scale can reveal the strengths and weaknesses of the regional ecological environment, which is of great practical significance for formulating regional ecological and environmentally sustainable development strategies.

Over the past decade, the rapid development of remote sensing technology, combined with the powerful spatial analysis function of GIS, has made up for the lack of meteorological data observation scale and provided the technical means for multi-scale and multi-temporal monitoring [9,10]. Most studies generally use Landsat images with a high spatial resolution. However, due to the limitation of the revisit period of Landsat satellites, the temporal resolution is low, and it is not possible to make long continuous observations on the ground over a large area [11]. The data of different years on the same date are vulnerable to climatic factors, such as precipitation and cloudiness [12]. The Moderate Resolution Imaging Spectroradiometer (MODIS) on board the Terra and Aqua satellites, which is designed for large-scale global dynamic monitoring, revisits every 1 to 2 days on average, has a higher precision temporal resolution, and has more significant advantages for large-scale, long time series observations [13]. In order to explore the significance of trend changes in time series, a series of trend change analysis methods have emerged as a result. The Mann–Kendall (M–K) nonparametric test can determine the significance level of the time series trend and has the characteristic of not being perturbed by outliers; the Theil–Sen slope is also insensitive to outliers and is more accurate than the results of non-robust simple linear regression [14]. The M–K test and Sen’s slope combination have been commonly applied to trend analysis in vegetation cover, wind speed, surface temperature, precipitation, and hydrology [15]. Related studies have generally used interannual and summer variation for trend variation analysis, but few studies have investigated long-term seasonal variation. The seasonal variation of LST is vital for managing crop spring planting and autumn harvesting, the variation in atmospheric dryness and humidity, and the magnitude of river runoff. Therefore, a comprehensive understanding of the long-term seasonal trends of LST is also essential.

The spatial distribution of high and low LST levels strongly influences regional climatic conditions. Previous studies show that the causes affecting LST changes are divided into three categories [16]: (1) land cover factors, including land use/land cover (LUCC) [17], land use intensity [18], land use landscape pattern [19,20], and vegetation cover [21]; (2) socio-economic factors, including anthropogenic heat and air pollution [22,23], population density [24], industrial layout [25], and energy consumption [26]; and (3) natural elements, including elevation [27], slope [28], precipitation [29], and light [30]. Numerous studies have shown that conditions such as a smaller density and shape of landscape pattern patches, higher vegetation cover, and more rivers and precipitation have a cooling effect on LST [31]. Areas with a high intensity of human activity, high population density, and low elevation have a warming impact on LST [32]. However, the applicability of these studies to arid regions needs to be further explored at this time.

Regarding the correlation analysis of LST with each influencing factor, traditional methods usually use linear methods, such as Pearson correlation analysis and ordinary least squares (OLS) [33,34]. Nevertheless, LST distribution is spatially heterogeneous, and traditional models tend to ignore these issues. The geodetector (GD) model has recently been widely used as a novel tool for exploring spatial differentiation and multifactorial drivers [35]. The model allows the independent detection of hierarchical structures, i.e., nonlinear relationships, and has the unique role of quantifying the force of the interaction of two independent variables on the dependent variable [36]. However, the GD model incorporates only the spatial information of the factors into the data processing system, and the presentation of the results is still global. Contrary to the GD model, the regression coefficient section of the multi-scale geo-weighted regression (MGWR) model has significant advantages for local regression analysis. The MGWR model is an optimized version of the geographically weighted regression (GWR) model, allowing the impact factor to vary with LST at different spatial scales, with various model results for each statistical unit, which is more intuitive and accurate for the interpretation of spatial heterogeneity in the degree of impact [37,38]. Therefore, this paper integrates the respective advantages of the GD model and MGWR model, which will have a more systematic explanatory power for LST spatial heterogeneity.

Urban clusters are advanced territorial spatial carriers of regional economic development, the degree of industrial agglomeration, and urbanization to a certain depth [39,40]. Most of the current Chinese urban agglomerations are located in the geographically advantageous eastern coastal areas, such as the Guangdong–Hong Kong–Macao–Great Bay Area urban agglomeration [41] and the Beijing–Tianjin–Hebei urban agglomeration [42]. The urban agglomeration on the northern slopes of the Tianshan Mountains, as the westernmost urban cluster in the arid and semi-arid regions of China, deep in the continent’s interior and rich in radiation and heat resources, has a typical oasis ecosystem [43]. By oasis, we mean that the warm air over its large-scale desert background matrix forms an inversion layer of “hot above and cold below” with the small-scale greenery through local circulation, constituting a relatively stable microclimate heterogeneous landscape and a gathering place for human activities [44]. In addition to population growth and urban sprawl, land desertification, soil erosion, and vegetation degradation have occurred frequently in recent years [45]. The fragile oasis ecosystem of the urban cluster on the northern slopes of the Tianshan Mountains is facing unprecedented challenges. Consequently, it is necessary to comprehensively understand the long-term seasonal trends of LST in the urban agglomeration on the northern slopes of the Tianshan Mountains based on their demographic, spatial, climatic, and topographical characteristics.

Based on this, this paper investigates the seasonal dynamics of LST and its driving forces in terms of spatial and temporal configurations and spatial composition, using the urban cluster on the northern slopes of the Tianshan Mountains in Northwest China as the study area. This study aims to (1) analyze the change in the LST trend over 18 years in the urban agglomeration on the northern slopes of the Tianshan Mountains and the future trend to provide countermeasures for preventing urban heat waves; (2) explore the spatial heterogeneity of LST and its influencing factors, and to provide a reference for subsequent related studies.

## 2. Study Area and Data Sources

### 2.1. Study Area

**Figure 1:**
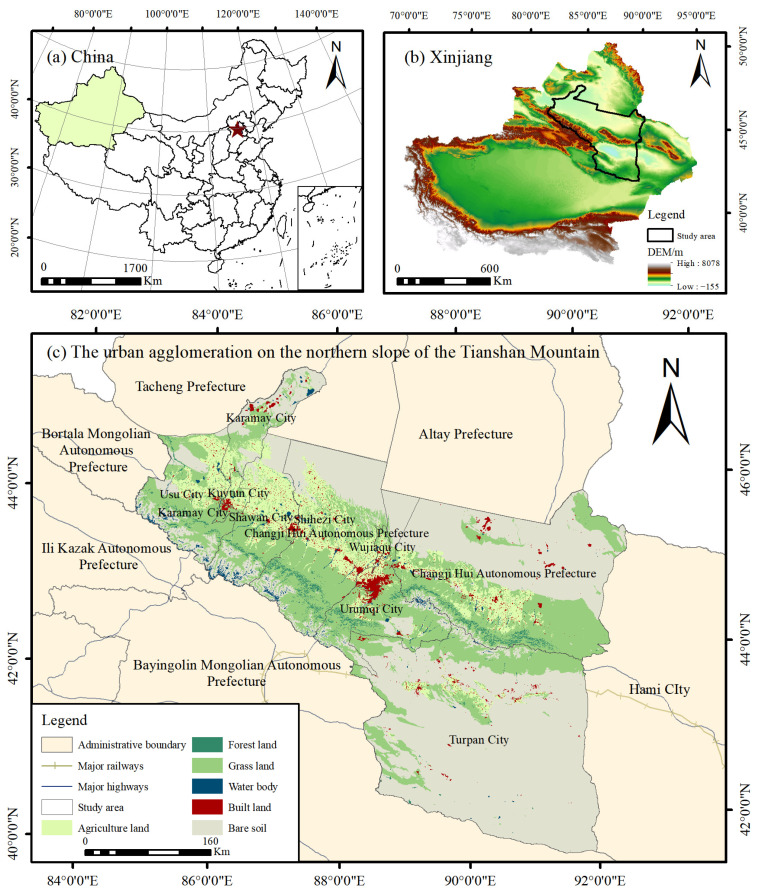
Location of the study area.

The urban agglomeration on the northern slopes of the Tianshan Mountains is an essential gateway to implementing the “Belt and Road” strategy. It is the region with the highest level of economic development in Xinjiang, China. It is located in the middle of the northern foot of the Tianshan Mountains and the southern part of the Junggar Basin, with a total area of 2.154 × 10^5^ km^2^; land use type dominated by unused land, grass land, arable land, and construction land; rich reserves of natural resources, such as oil and natural gas; and a total population of 5.9171 × 10^6^ people in 2018, accounting for one fifth of the total population of Xinjiang, and it is a driving force for the development of towns and economic construction in the Xinjiang region hub (Figure 1).

### 2.2. Data Sources

#### 2.2.1. LST Data

NASA launched the Aqua satellite in April 2002, and related studies have shown that the LST products from the Aqua satellite inversion are more accurate than those from the Terra satellite [46]. MODIS is combined with the Visible Infrared Imaging Radiometer Suite (VIIRS) to obtain a 1 km resolution LST using a generalized split-window algorithm inversion, which is capable of continuous superimposed observations [47]. Therefore, the MYD11A2 LST product was verified by field measurements and produced only a <0.5 K deviation [48]. In this study, we used the MODIS Aqua LST 8-day synthetic product (MYD11A2) daytime data from 2003 to 2020 with a spatial resolution of 1 km, derived from the Google Earth Engine (GEE) (https://earthengine.google.com) (accessed on 27 March 2022).

Based on the GEE platform, first, the function to convert Kelvin degrees to Celsius degrees was created; second, a total of 828 images over 18 years were mean-composed on a seasonal scale, resulting in 72 images. The seasons are divided into spring from March to May, summer from June to August, autumn from September to November, and winter from December to February. Data often have missing values due to weather or cloudy conditions, sensor errors, track scan vacancies, etc. After repeated experiments, the average interpolation of 3 × 3 pixels, 9 × 9 pixels, or 11 × 11 pixels was used to fill the data null for images with different degrees of vacancy.

#### 2.2.2. Other Data

In this paper, we selected DEM data and extracted slope and aspect data from them; MODIS NDVI product (MOD13Q1) was used to illustrate vegetation cover; nighttime light (NTL) was used, using DMSP/OLS and NPP/VIIRS data, respectively, to indicate population distribution. The above data are also from the GEE platform (https://earthengine.google.com) (accessed on 10 April 2022). This paper set the spatial resolution output format to 1 km for data consistency. The land use data were obtained from the Resource Environment Science and Data Center (https://www.resdc.cn/) (accessed on 13 April 2022) at a spatial resolution of 30 m. The land use types in the study area were classified into six categories: agricultural land, forest land, grass land, water body, built land, and bare soil. The month-by-month precipitation (P) and air temperature data were obtained from the Tibetan Plateau Science Data Center (https://data.tpdc.ac.cn/zh-hans/) (accessed on 16 April 2022) with a spatial resolution of 1 km.

## 3. Research Methods

**Figure 2:**
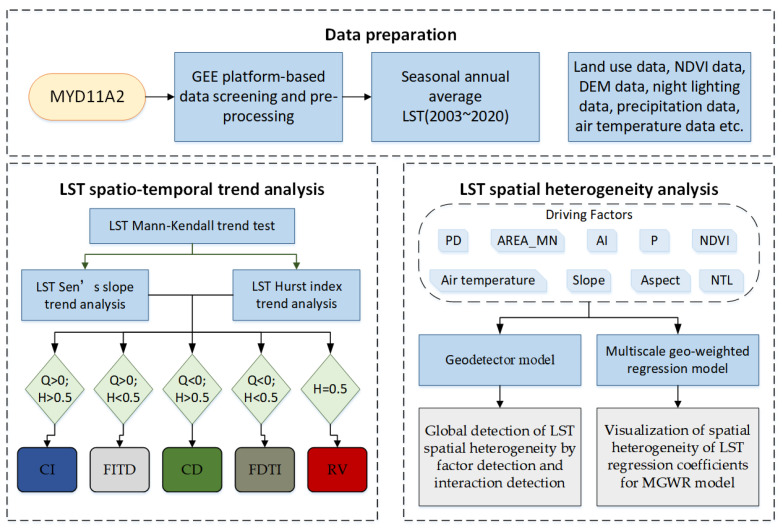
Research framework.

First, this paper processed the data based on the GEE platform, and we used the mean-standard deviation method to classify the LSTs. Second, the characteristics and trends of seasonal LST spatial and temporal distribution of the urban agglomeration on the northern slopes of the Tianshan Mountains from 2003 to 2020 were quantified using trend analysis, such as the M–K test, Sen slope, and Hurst index. Third, the GD and MGWR models were used to analyze the effects of land use landscape pattern, slope, slope orientation, vegetation, precipitation, air temperature, and population on LST and to find the influencing factors that can significantly mitigate LST in arid areas. The research framework is shown in Figure 2.

### 3.1. LST Level Classification

This paper used the mean–standard deviation method to classify the temperature classes into extremely low temperature (ELT), low temperature (LT), medium temperature (MT), high temperature (HT), and extremely high temperature (EHT) (Table 1). The mean-standard deviation method works best for datasets with normal distribution and can maintain the robustness of LST after classification [49].

### 3.2. Trend Change Analysis

#### 3.2.1. Mann–Kendall Test

The Mann–Kendall (M–K) test is a nonparametric trend test, which is often used to determine the significance of changes in temperature, precipitation, and other factors over a long time series, mainly through the standard statistic *Z* and confidence level α to determine the significance of the data [50,51]. Suppose the LST time series are $x_{0},\;x_{1},\;x_{2},\cdots ,x_{n}$, and they are independently identically distributed, for any i<j<n and i≠j, the statistic *S* is calculated:

(1) $$S=\sum_{i=1}^{n-1}\sum_{j=i+1}^{n}\mathrm{sgn}\left(x_{j}-x_{i}\right)$$

where sgn() is the symbolic function whose function equation is:

(2) $$\mathrm{sgn}\left(x_{j}-x_{i}\right)=\left\{\begin{array}{cc} 1, & x_{j}-x_{i}>0\\ 0, & x_{j}-x_{i}=0\\ -1, & x_{j}-x_{i}<0 \end{array}\right\}$$

*S* obeys the normal distribution and (*n* > 0), and constructs the standard normal distribution statistic *Z*:

(3) $$Z=\left\{\begin{array}{cc} \frac{S-1}{\sqrt{\mathrm{Var}\left(S\right)}}, & S>0\\ \;\;\;\;\;\;\;\;0\;\;\;\;\;\;\;, & S=0\\ \frac{S+1}{\sqrt{\mathrm{Var}\left(S\right)}}, & S<0 \end{array}\right\}$$

where Var(S) is the variance of *S*:

(4) Var(S)=n(n−1)(2n+5)/18

When taking 0.95 as the confidence level, the value of *Z* is 1.96, then *Z* ≥ 1.96 is a significant rise (SR); 0 < *Z* < 1.96 is a general rise (GR); *Z* = 0 is not significant (NS); 1.96 < *Z* < 0 is a general decline (GDL); and *Z* ≤ −1.96 is a significant decline (SDL).

#### 3.2.2. Sen’s Slope

In contrast to traditional slope analysis, Sen’s slope is not disturbed by a few abnormal values and is widely used for the trend analysis of time series [52,53]. The basic principle is as follows:

(5) $$Q=median\frac{LST_{j}-LST_{i}}{j-i}\;\;\;\;\;1<i<j<n$$

where *i* and *j* are the number of time series; $LST_{i}$ and $LST_{j}$ are the LST values of the *i*-th and *j*-th time series, respectively; and the slope *Q* > 0 indicates a warming trend, while *Q* < 0 indicates a cooling trend.

#### 3.2.3. Hurst Index

Hurst originally proposed the Hurst index in 1951 to reveal the long-term temporal correlation and dependence of time series data. [54]. Additionally, combined with the slope, it can predict future trends in change. A rescaled polar difference (*R*/*S*) analysis method is commonly used [55], the basic principle of which is as follows.

Define the time series $\left\{LST_{\left(t\right)}\right\},t=1,2,\cdots ,n$ as the mean series.

(6) $${\bar{LST}}_{\left(T\right)}=\frac{1}{T}\sum_{t=1}^{T}LST_{\left(T\right)}\;\;\;\;\;T=1,2,\cdots ,n$$

Cumulative deviation:

(7) $$X_{\left(t,T\right)}=\sum_{t=1}^{t}\left(LST_{\left(t\right)}-{\bar{LST}}_{\left(T\right)}\right)$$

Extreme difference:

(8) $$R_{\left(t\right)}=max_{1\leq t\leq T}X_{\left(t,T\right)}-min_{1\leq t\leq T}X_{\left(t,T\right)}$$

Standard deviation:

(9) $$S_{\left(t\right)}={\left[\frac{1}{T}\sum_{t}^{T}{\left(LST_{\left(t\right)}-LST_{T}\right)}^{2}\right]}^{\frac{1}{2}}$$

where 1 ≤ *t* ≤ *T*, *T* = 1, 2,⋯, *n*; the $R_{\left(t\right)}/S_{\left(t\right)}≅R/S$ ratio is obtained from the extreme difference and standard deviation; if $R/S\propto T^{H}$, *H* is the Hurst index, which indicates the presence of the Hurst phenomenon in the time series LST.

When 0 < *H* < 0.5, this indicates that the LST time series has anti-continuity, i.e., the future change is opposite to the past; when *H* = 0.5, this indicates that the LST time series changes randomly and the future change is independent of the past; 0.5 < *H* < 1 indicates that the future change in LST time series is consistent with the past and has continuity. The data processing for the above trend change analysis method is based on the programing software R4.1.2 (Oakland, New Zealand).

### 3.3. Geodetector Model

The geodetector (GD) model is a nonlinear statistical model proposed and created by Wang et al. (2012), which has the feature of detecting the spatial heterogeneity of influencing factors and can achieve the independent detection of different influencing factors [56]. The GD model includes four modules: factor detection, interaction detection, risk detection, and ecological detection, and, in this study, only factor detection and interaction detection are used. GD model detection requires data discretization, so most studies commonly use the natural interruption point method to classify continuous-type data uniformly and then derive the final results in the GD program (http://www.geodetector.cn/) (accessed on 2 May 2022). In contrast, the R-based “GD” package uses optimal parameters to discretize the data before modeling, and each datum has specific parameters, and its detection results are more realistic [36]. Therefore, this paper uses R4.1.2 for data processing.

#### 3.3.1. Factor Detector

Factor detection uses *q* values to express the degree of explanation of different influence factors on the spatial heterogeneity of LST. The value of *q* is taken as [0, 1], and the higher the value, the higher the explanatory power of the independent variable on the dependent variable LST and vice versa. The equation is as follows:

(10) $$q=1-\left(\sum_{h1}^{L}N_{h}\sigma_{h}^{2}/N\sigma^{2}\right)$$

where *h* = 1,…, *L* is the stratification of the LST or impact factor, i.e., classification or partition; *N_h_* and *N* are the number of cells in layer *h* and the whole area, respectively; and $\sigma_{h}^{2}$ and *σ*^2^ are the variances of the LST in layer *h* and the entire region, respectively.

#### 3.3.2. Interaction Detector

Interaction detection identifies whether two influences, when acting together, strengthen or weaken the explanatory power of surface temperature. The identification process calculates the q-values of two factors: q (X1) and q (X2). The value of q for which they are superimposed is calculated, i.e., q (X1∩X2), q (X1), q (X2), and q (X1∩X2) are compared. When q (X1∩X2) < Min (q (X1), q (X2)), the interaction detection result is nonlinear–weaken; when Min (q (X1), q (X2)) < q (X1∩X2) < Max (q (X1), q (X2)), the interaction detection result is uni-variable weaken; when q (X1∩X2) > Max (q (X1), q (X2)), the interaction detection result is bi-variable enhance; when q (X1∩X2) = q (X1) + q (X2), the interaction detection results are independent; when q (X1∩X2) > q (X1) + q (X2), the interaction detection result is nonlinear–enhance.

#### 3.3.3. Data Preparation

The landscape index is the coalescence of landscape pattern information, reflecting the overall composition and characteristic structure of landscape elements [57]. This paper selected three typical land use landscape indices based on land use data from six land use types: patch density (PD) to characterize landscape porosity and reveal the degree of fragmentation of heterogeneous landscapes, average patch area (AREA_MN) to reflect the size of patches in landscape types, and the aggregation index (AI) to characterize the connectivity and dispersion among heterogeneous landscape patches [58]. In this way, we can explore the spatial relationship between land use landscape patterns and LST. Due to the large size of the study area, this paper selected a 10 km × 10 km grid to divide the study area into 3910 subgrids. The landscape index of each subgrid was calculated separately using Fragstats 4.2 software (Corvallis, OR, USA). In addition, it also includes a slope, aspect, NDVI, air temperature, nighttime lighting, and precipitation data, a total of 9 influencing factors.

### 3.4. MGWR Model

The MGWR model is based on the concept of GWR model construction, using Gaussian kernel functions to spatially weight the influence elements, and, using the Akaike information criterion (AIC) to determine the optimal choice of bandwidth for each influence element based on weighted least squares combined with generalized weighted model estimation to fit the algorithm, each influence element has its own spatial smoothing level, which can reduce the estimation error and enhance the accuracy of the model [59,60]. The basic principle is as follows:

(11) $$y_{i}=\beta_{0}\left(u_{i}+v_{i}\right)+\sum_{j=1}^{k}\beta_{bwj}\left(u_{i}+v_{i}\right)x_{ij}+\varepsilon_{i}$$

where $\left(u_{i}+v_{i}\right)$ is the center co-ordinate of the position *i* of LST; bwj is the bandwidth calculated using the *j*th regression coefficient; $\beta_{0}$ is the intercept term; $\varepsilon_{i}$ is the error term; and $\beta_{bwj}\left(u_{i}+v_{i}\right)$ is the regression coefficient of the *j*th influencing element at position *i* of LST. The operation process of the above model is carried out in MGWR 2. 2 software (Phoenix, AZ, USA), where the type of spatial kernel function selected during the model run is Bisquare, the bandwidth search type is Golden, and the initialization type of model parameters is GWR estimation as the initial estimated model.

## 4. Results

### 4.1. LST Spatiotemporal Variation Analysis

**Figure 3:**
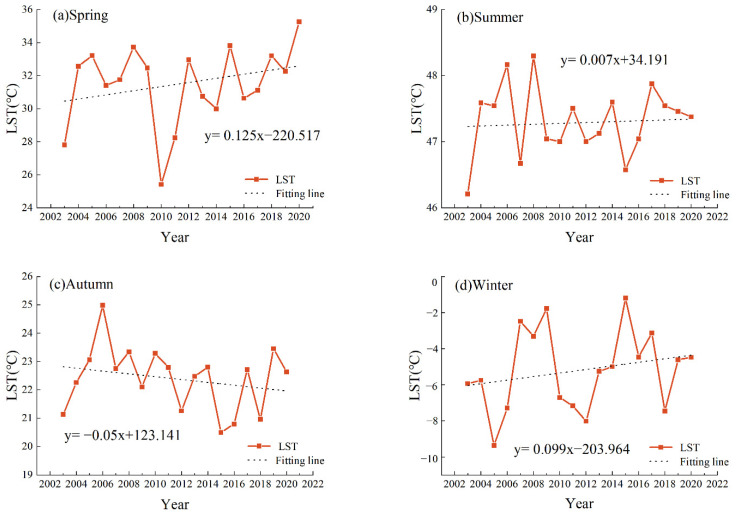
Seasonal LST change from 2003 to 2020.

**Figure 4:**
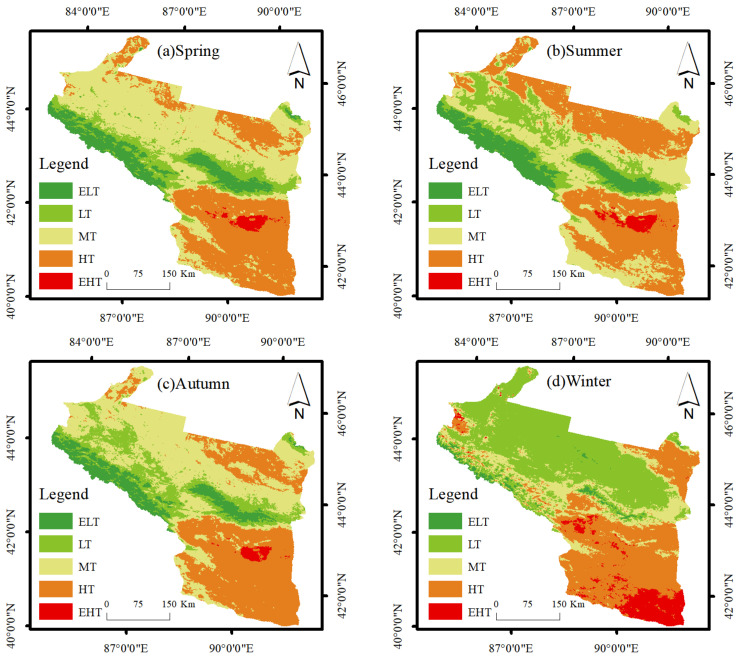
Average LST by season from 2003 to 2020.

Based on MYD11A1 data, this paper used the mean–standard deviation method to classify LST and obtain the seasonal LST of the urban agglomeration on the northern slope of Tianshan Mountain from 2003 to 2020 (Figure 3 and Figure 4). We tested the time trend of the linear regression for significance, and the results yielded *p* < 0.05, which is statistically significant. The annual average LST in spring was 31.53 °C, with a warming rate of 0.125 °C·a^−1^, the most significant trend compared with other seasons, with extreme weather events occurring in 2010, which is proximately 4 °C lower than the mean LST in spring. The annual average summer LST was 47.29 °C, with a warming rate of 0.007 °C·a^−1^ and a relatively flat trend, the highest value being 48.29 °C in 2008 and the lowest value being 46.21 °C in 2003, with the difference between high and low temperatures being around 2 °C. Autumn is the transitional season of winter, and most of the region is dominated by cooling, with an annual average LST of 22.38 °C and a relatively stable trend change of −0.05 °C·a^−1^, with a maximum value of 24.98 °C in 2006. The average yearly winter LST was −5.20 °C, with a warming rate of 0.099 °C·a^−1^.

Bare soil has desert as the primary land use type; the surface heats up rapidly, has a strong heat collection capacity, and occupies the most expansive area in the arid zone. Therefore, HT types occupy a large proportion throughout the year (Table 2, Figure 4). EHT is mainly distributed in the Turpan basin, known as the “furnace of China”, with an average LST of up to approximately 65 °C in summer. The largest area occupied by medium temperature was 42.45% and 38.69% in spring and autumn, respectively, which was due to the reduction in agricultural land area. This shows that agricultural land has the most significant cooling effect on LST.

### 4.2. Trend Change Analysis and Forecast

#### 4.2.1. Trend Change Analysis

**Figure 5:**
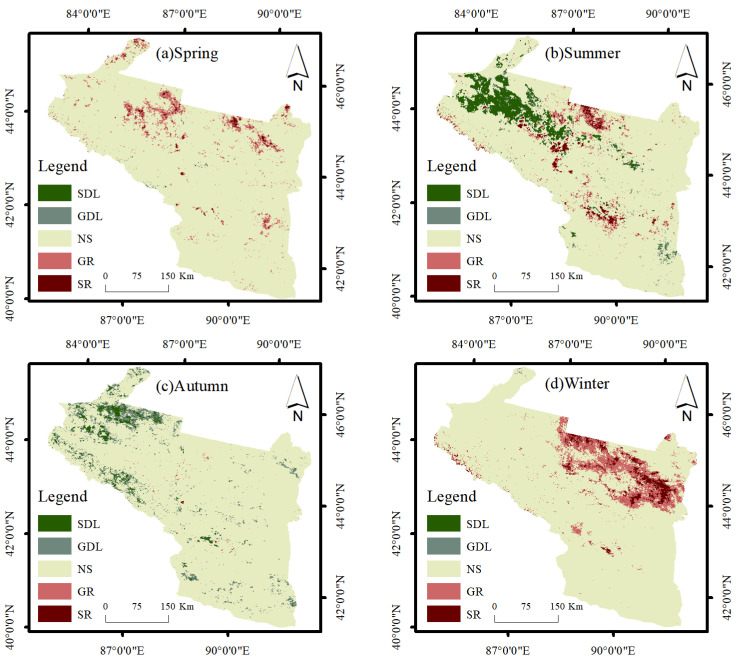
Average LST M–K test for 2003 to 2020.

From the M–K nonparametric test (Table 3, Figure 5), the LST in spring showed an overall increasing trend. Bare soils are the first to warm up, so GR and SR are mainly located on bare land and some agricultural land. In summer, SDL and GDL were mainly concentrated in agricultural land, accounting for 9.1% of the total area due to the higher vegetation coverage in agricultural land and the good cooling effect; GR and SR were mainly concentrated in some built land and bare land. In addition, perennial precipitation is scarce in arid areas, and the vegetation of the mountains on the northern slopes of Tianshan is mainly desert grass land with a low level of vegetation cover; therefore, the cooling effect of grass land is not as significant as that of agricultural land. The warming of built land is due to the temperature increase caused by the rise in the proportion of impervious surfaces brought about by urban development. The SDL and GDL had the highest percentage in autumn, with a decreasing trend in temperature, mainly in woodlands, some agricultural fields, and bare soils. While the LST showed a warming trend in winter, the GR and SR accounted for 12.65%, mainly concentrated in bare soil, indicating that the region was the first to warm up during the transition from winter to spring.

#### 4.2.2. Predicted Temporal Trends in LST

**Figure 6:**
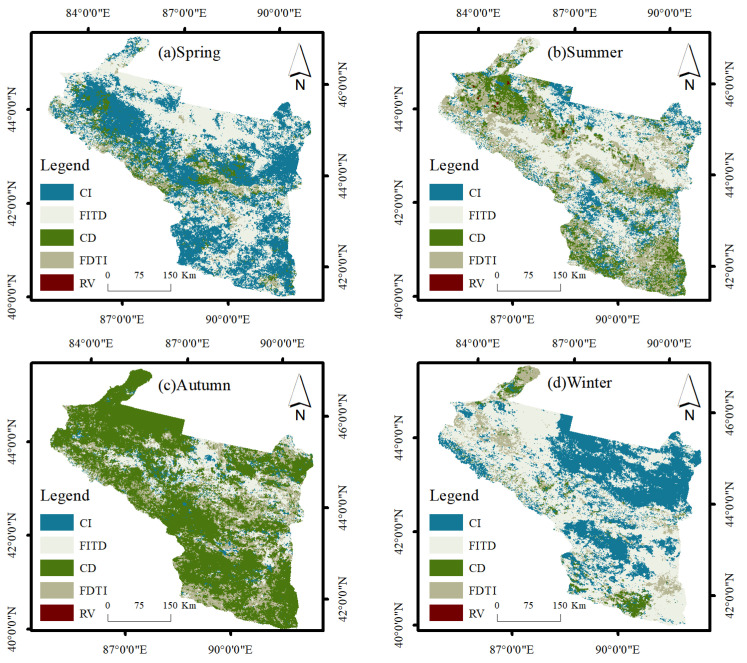
LST direction change prediction.

This paper used the Hurst index to visually analyze the long-term temporal correlation of the LST, superimposed with Sen’s slope, to predict the future temperature trend (Figure 6, Table 4). Q > 0 and H > 0.5, then the future direction of surface temperature shows a continuous improvement (CI); Q > 0 and H < 0.5, there is from increase to decrease (FITD); when Q < 0 and H > 0.5, there is continuous degradation (CD); when Q < 0 and H < 0.5, there is from decrease to increase (FDTI); when H = 0.5, it indicates random variations (RV).

In spring, the temperature of the LST will rise successively in different regions in the future. CI and FITD accounted for 87.41% of the total area of the study area. Nevertheless, the warming trend in the FITD area began to slow down in the later period, and the place was mainly concentrated in the bare land, indicating that the bare land LST will tend to balance when the temperature reaches a certain level. The warming trend of some glaciers and rivers in spring is not significant. In summer, FITD accounts for 42.75% of the area of the study area, and the warming trend is not as substantial as that in spring. The future warming/cooling trend is strongly affected by land use. In autumn, CD accounted for 61.71% of the total area, the heat collection capacity of some bare land was strong, and the cooling trend had not yet appeared. Except for deserts, the LST is maintained below 0 °C in winter, and there is an inevitable warming trend during the transition to spring, consistent with the M–K test results.

### 4.3. LST Impact Factor Geographic Detection

In order to explore the spatial heterogeneity of LST and its response to temporal and spatial changes, this paper selects nine types of influencing factors to examine the degree of influence on LST based on the seasonal LST in 2003, 2010, and 2020, including PD, AREA_MN, AI, P, air temperature, NDVI, slope, aspect, and NTL. The results of the factor tests (Table 5) show that the q-values of each influence factor, due to the large size of the study area, fluctuate less in the range of time scales. The q-values of P, air temperature, and NDVI were higher in all seasons and years, indicating that precipitation, air temperature, and vegetation had the most decisive influence on LST, while the q-values of slope and NTL were the lowest, indicating that the slope factor of the terrain and the population distribution had a weak influence on LST. Among them, the q-values of P and air temperature vary with the seasons. p has the most substantial effect in summer, and air temperature has only a slightly weaker effect in autumn. In addition, the comparison shows that the influence of the summer landscape pattern on LST in 2020 is more evident than in other seasons, and the q-values of each influencing factor on summer LST are P > air temperature > NDVI > PD > AI > AREA_MN > slope > aspect > NTL.

**Figure 7:**
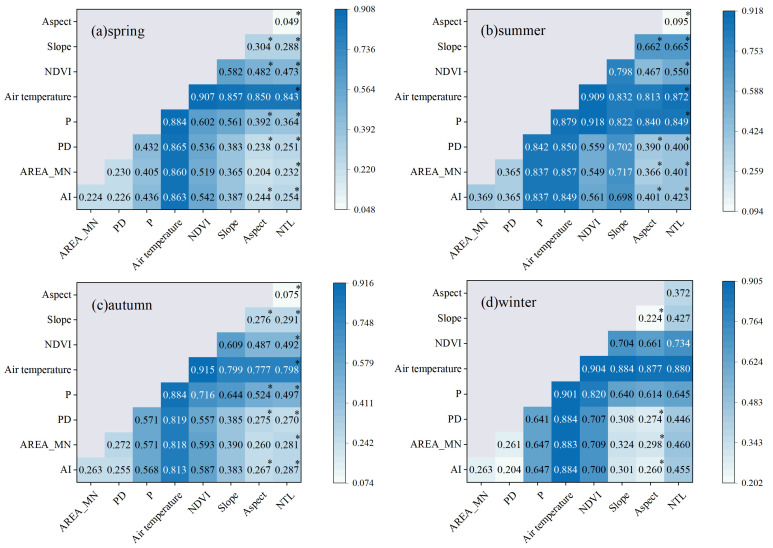
Interaction detection diagram in 2020 (* represents nonlinear–enhance; unmarked values indicate bi-variable enhance).

Interaction probes were performed on the different influencing factors in 2020 (Figure 7) to determine the degree of explanation of LST when the two influencing factors interacted. The dominant interaction factor in each season was air temperature∩NDVI, with q values of 0.907, 0.909, 0.915, and 0.914, respectively, followed by P∩air temperature and P∩NDVI. In summer, the q-values of most influencing factors are more significant than 0.5, and the interaction force is the most powerful. As can be seen from the figure, comparing the interaction force of two factors with the single factor force, the interaction has more influence on LST, which implies that the spatial differentiation of LST is not determined by one factor but is driven by multiple elements together. In particular, the interaction of aspect and NTL with each element was mainly nonlinear–enhance, with the lowest single factor q value of aspect and NTL in all seasons, but reached a double effect with the interaction, which greatly enhanced the influence on LST.

### 4.4. Drivers of LST and Analysis of Spatial Differences

In order to deeply analyze the drivers of LST changes in detail, based on the GD model detection results, this paper selects relevant data in the summer of 2020. It uses the statistical advantage that the regression coefficients of the MGWR model are local regressions to reveal the influence of its drivers on the local LST of the urban agglomeration on the northern slopes of the Tianshan Mountains. The MGWR model determines the optimal bandwidth for each variable, which is advantageous for univariate analysis. The magnitude of the regression coefficient can find the critical factor influencing the local LST, and the larger the absolute value of the regression coefficient (i.e., the high-value area), the greater the degree of influence on the LST. Before conducting the regression analysis, to check whether there is any multicollinearity among the explanatory variables, this study used SPSS 26.0 software (Armonk, NY, USA) to test each explanatory variable for the co-linearity test and obtain the variance inflation factor (VIF). The test results showed that the VIF values of PD, AREA_MN, AI, P, air temperature, NDVI, slope, aspect, and NTL were 3.175, 2.024, 4.006, 9.261, 9.402, 1.216, 3.801, 1.028, and 1.034, respectively, all of which are less than 10, indicating no significant effect of multicollinearity among the explanatory variables. The regression analysis can be carried out in the next step. The corrected decidability coefficient (Adj-R^2^) represents the degree of explanation of the dependent variable by the influencing factor; Log-likelihood (LIK), the Akaike information criterion (AIC), and residual sum of squares (RSS) help evaluate the performance of the model, where larger Adj-R^2^ and LIK, and smaller AIC and RSS indicate a better fit of the model. From the analysis of the global regression results (Table 6), the R^2^ is 0.978, and all coefficients of MGWR are significant overall, which can be fully used to assess the relationship between LST and impact factors.

**Figure 8:**
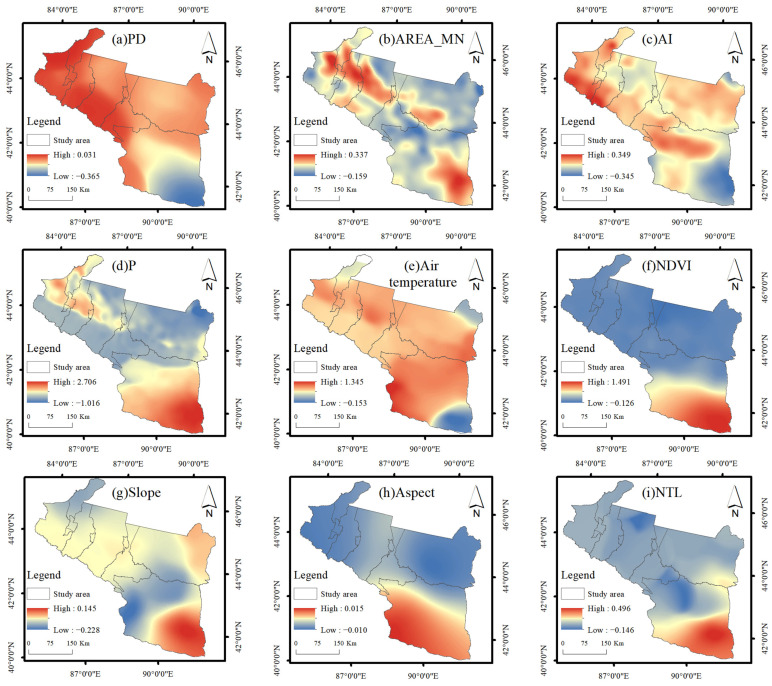
Spatial distribution of regression coefficients of LST influence factor patterns.

It can be seen from Figure 8 that the influence of PD on LST is primarily positive, and negatively correlated in some parts of Turpan. The impact area of AREA_MN is highly similar to the land use status, with the high-value areas mainly located on agricultural land, forest land, and built land and the low-value areas on bare soil. While AI effect on LST is opposite to AREA_MN, the high-value area is mainly distributed on bare soil. The impact of P on LST is primarily negative, with high-value areas concentrated in the Turpan region. The effect of air temperature on LST is basically a warming effect within the study area. NDVI is mainly a cooling effect, with nearly no vegetation cover at the location of the high-value area. The influence of slope on LST mainly affects Urumqi City, Wujiaqu City, Changji Hui Autonomous Prefecture, Shihezi City, and Shawan City. This area is located in the Tarim Basin, with small terrain fluctuations and a wide range of influence. The influence of aspect and NTL on LST is weak, and the high-value area is mainly located in the Turpan Basin.

## 5. Discussion

### 5.1. Comparative Analysis of LST Differences

The distribution of the surface thermal environment is a response to changes in surface sensible heat fluxes, which has positive implications for regional crop growth, ecological quality assessment, local climate change, etc. [61]. The arid and semi-arid areas form a unique landscape unit, which is caused by the difference between the “furnace” desert and the underlying surface of other landscape types and the uneven distribution of surface heat [62]. Compared with the urban agglomeration along the Yellow River in Ningxia and the urban agglomeration in Hubao-Eyu, which are also located in arid and semi-arid regions, the urban agglomeration on the northern slopes of the Tianshan Mountains has the highest drought level. It is strongly influenced by the Siberian winter monsoon and continental air mass, resulting in large temperature fluctuations, high summer temperatures, severe winters, and sparse rainfall throughout the year, with distinct seasons [63,64]. From a time point of view, the alternation of the four seasons makes the interaction of cold and warm currents quite frequent. The seasonal LST will directly affect the sowing and growth of cash crops, which is of great importance for agricultural work, the quality of agricultural products, economic construction, and agricultural development [65]. In addition to climatic factors, in terms of spatial pattern, the urban agglomeration on the northern slope of the Tianshan Mountains also has a unique structural layout. The average altitude of the Tianshan Mountains is 4000 m, is covered with ice and snow all year round, and the temperature is maintained below 0 °C. However, the Turpan Basin has an altitude of −154.31 m, which is the region with the lowest altitude and the highest summer temperature in China. Therefore, the variation in LST is also quite different in the vertical direction [66,67]. This mountain–basin interaction constitutes the characteristic oasis ecosystem of the urban agglomeration on the northern slope of the Tianshan Mountains. China is placing more and more emphasis on environmental protection initiatives, and the 14th Five-Year Plan focuses on environmental management issues. However, the steady recovery of the oasis ecosystem will take a long time.

### 5.2. Temporal Variation Trend of LST and Its Spatial Heterogeneity

Over time, the LST distribution of urban agglomerations is the result of a combination of factors, such as changes in land use structure, increase in impervious surfaces, such as concrete structures and gravel density due to population surge, atmospheric aerosols, increase in CO_2_ emissions, population density, changes in climatic variables, slope, and elevation of topography [68]. Based on the time series products of LST, this study focuses on the seasonal trends of LST in the urban agglomeration on the northern slopes of the Tianshan Mountains and analyzes the causes causing LST changes. The study found that the seasonal LST is mainly characterized by fluctuating changes, with an overall upward trend but a downward trend from autumn to winter, consistent with previous studies’ results [69]. The reason for the weakening of the LST from autumn to winter is usually that the evaporative cooling effect is more robust in the vegetated area than in winter. In addition, here, there is variability in the influence of different influencing factors on LST. Chief among these are climate indicators, such as air temperature and precipitation, which are also critical to global climate change. The negative correlation between LST and precipitation showed seasonal characteristics [29]. High temperatures in summer caused soil water evaporation and indirectly affected precipitation. Therefore, the relationship between LST and precipitation was more sensitive in summer than in other seasons. The relationship between air temperature and LST is in a state of long-term interaction. Therefore, their strong correlations do not vary with time and seasonal changes. Under arid conditions, LST was mainly positively correlated with air temperature. However, LST was negatively correlated with air temperature in some cities with less urban–rural vegetation differences and low vegetation cover [70]. Under the conditions of global climate change, extreme heat waves and droughts with little rain will be more frequent, vegetation plays a neutralizing and regulating role in this link, and increasing urban greening construction can, to a certain extent, reduce the adverse effects brought by global climate change.

### 5.3. Analysis of Landscape Pattern and LST Spatial Relationship

Regarding the positive and negative effects of quantitative landscape metrics on the impact of LST, Xiang et al. (2022) noted that AI and LST were always negatively correlated, while PD and LST were positively correlated [19]. While Liu et al. (2020) found that a higher PD would lead to a lower LST [71]. Peng et al. (2016) believed that AREA_MN had a strong negative correlation with LST, and AI had a positive correlation with LST [72]. Using a semi-parametric geographically weighted regression (SGWR) model, Li et al. (2017) found that, for areas with a highly intense of heat island effect, the density and degree of aggregation of plaques negatively affected LST [73]. The effects of patch density and patch area vary spatially with the fragmentation of heat island intensity. Therefore, the landscape patterns affecting the positive and negative effects of LST vary across geographic regions [74]. For example, on the one hand, the transpiration of vegetation and the shading of tree canopies have a cooling effect, and this will limit the cooling effect in areas with high fragmentation and poor connectivity of the green landscape [75]; on the other hand, in areas with a high fragmentation of patches, the shadows derived from vegetation on surrounding features have the probability of expanding and causing local temperature reduction [76]. The influence of landscape patterns on LST is unstable, and multiple scenarios need to be considered when exploring the correlation between landscape elements and LST. Areas with high connectivity of bare soil patches have higher LST, and regions with high connectivity of green land patches have lower LST, with regional distribution characteristics; but, as a whole, AI and AREA_MN are negatively correlated with LST, and PD is positively correlated with LST [62]. Therefore, in order to maintain the sustainable development of oasis ecosystems, attention needs to be paid to the construction of green and blue landscapes, which are essential for oasis ecological restoration, biodiversity, and the suitability of the human living environment.

### 5.4. Limitations and Future Perspectives

This paper explores the spatiotemporal variation in long time series LST and its driver analysis in the urban agglomeration on the northern slopes of the Tianshan Mountains but there are still some limitations. First of all, as one of the 19 urban agglomerations in China, the urban agglomeration on the northern slopes of the Tianshan Mountains has a smaller distribution of towns and construction land area due to its geographical location and topography, and its population density and per capita emissions, as well as its contribution to LST, are not as significant as those of other urban agglomerations.

Secondly, LST inversion is vulnerable to the constraining effects of the spatial and temporal scales of remote sensing images; using high spatial resolution sensors, such as Landsat and Sentinel data, etc., the feature recognition of surface observation is highly accurate and produces a minimal error but is limited by its orbit revisit period, transit time, and atmospheric quality, which makes it difficult to acquire continuous data. MODIS remote sensing image has a wide observation range, and its time resolution is high; however, its lower spatial resolution compared with high-resolution satellites is an unavoidable problem [77]. Therefore, detection and analysis using high-precision images for large-scale LST are essential in future work. Hutengs et al. (2016) used remote sensing images with a low spatial and temporal resolution for downscaling studies to obtain image data with both high temporal and spatial resolutions, which has become an essential direction for current LST research [78].

Thirdly, the nighttime lighting index is chosen as the socioeconomic factor in this paper, but the experiment shows that the effect of this factor on LST is not significant. Related studies have shown that population distribution, population density, etc., have limited impacts on LST, and the dominant characteristic of human influence on LST changes is anthropogenic thermal emissions; therefore, in future studies, there is a need to consider the anthropogenic thermal effect and the impact of GDP development on the socio–economic aspects [79].

Finally, by comparing the four seasons, the summer landscape pattern had the most significant effect on LST. Therefore, this paper selected only summer data for regression analysis; however, interannual and seasonal variation in LST in arid zones warrants further exploration in future studies. A comprehensive understanding of the mechanisms of LST variability is essential for preventing climate extremes and maintaining environmental quality.

## 6. Conclusions

(1) The average spring temperature of the urban agglomeration on the northern slopes of the Tianshan Mountains from 2003 to 2020 was 31.53 °C, with a slope of 0.125 °C·a^−1^; the average summer temperature was 47.29 °C, with a slope of 0.007 °C·a^−1^; the average autumn temperature was 22.38 °C, with a slope of −0.05 °C·a^−1^; and the average winter temperature was −5.20 °C, with a slope of 0.099 °C·a^−1^. EHT and HT are mainly distributed in bare land; MT and LT are primarily distributed in agricultural land, grass land, and built land; ELT is mainly distributed in forest land and water bodies.
(2) Using the Mann–Kendall test, Sen’s slope, and Hurst index, we analyzed the trend of LST changes and the future development direction of the urban agglomeration on the northern slopes of the Tianshan Mountains. The results showed that LST showed an increasing trend in spring, summer, and winter and a decreasing trend in autumn; bare land and grass land LST warmed more rapidly. Agricultural land LST had a significant cooling effect. In terms of future development, the pre-mountain basin was the first to warm up in spring, with CI occupying a more extensive area; the transition from summer to autumn FITD dominated; LST continued to degrade in autumn, with CD accounting for 61.71% of the total study area; and LST continued to rise in winter, with bare land being the first to warm up in the transition from winter to spring.
(3) The results of geodetector factor detection showed that air temperature and precipitation had the most significant effects on LST, and the magnitude of the q-value of each influence factor in summer was P > air temperature > NDVI > PD > AI > AREA_MN > slope > aspect > NTL. The interaction detection results show that air temperature∩NDVI, P∩air temperature, and P∩NDVI have the most substantial effect on LST.
(4) The MGWR model was used to analyze the relationship between each influence factor in space and local LST. The effects of PD, air temperature, and slope on LST were mainly positive, AREA_MN, AI, P, and NDVI were primarily negative, and the impact of aspect and NTL on LST were relatively weak.

## Figures and Tables

**Table 1:** LST classification level table.

LST Level	Classification Range	Spring/°C	Summer/°C	Autumn/°C	Winter/°C
ELT	T < μ − 1.5 std	−11.97~16.41	−0.33~27.09	−14.99~10.58	−25.38~−12.03
LT	μ − 1.5 std ≤ T < μ − 0.5 std	16.41~25.79	27.45~38.45	10.58~18.53	−12.03~−4.96
MT	μ − 0.5 std ≤ T < μ + 0.5 std	25.79~35.17	38.45~49.81	18.53~26.48	−4.96~2.12
HT	μ + 0.5 std ≤ T < μ + 1.5 std	35.17~44.55	49.81~61.17	26.48~34.43	2.12~9.19
EHT	T > μ + 1.5 std	44.55~50.90	61.17~68.21	34.43~38.54	9.19~14.66

T is the LST value, µ is the mean, and std is the standard deviation.

**Table 2:** Area of each grade of LST and its proportion.

Level	Spring		Summer		Autumn		Winter	
	Area km^2^	Proportion %	Area km^2^	Proportion %	Area km^2^	Proportion %	Area km^2^	Proportion %
ELT	17,343.9	9.94	19,106.1	10.95	15,728.4	9.01	3131.1	1.79
LT	20,399.4	11.69	28,377.0	16.26	28,169.1	16.14	72,622.8	41.60
MT	74,097.0	42.45	55,137.6	31.59	67,531.5	38.69	29,198.7	16.73
HT	60,199.2	34.49	68,825.7	39.43	61,272.9	35.10	57,188.7	32.76
EHT	2522.7	1.45	3115.8	1.78	1860.3	1.07	12,420.9	7.12

**Table 3:** Statistical table of changes in M–K trends.

Season	SDL	GDL	NS	GR	SR
Spring	0.03 **	0.14 *	94.15	4.88 *	0.79 **
Summer	6.82 **	2.28 *	85.20	3.84 *	1.87 **
Autumn	2.30 **	6.15 *	91.34	0.18 *	0.04 **
Winter	0.00 **	0.05 *	87.29	9.94 *	2.71 **

** means passing the confidence test of 0.01; * means passing the confidence test of 0.05.

**Table 4:** LST future change trend statistics table.

Season	CI/%	FITD/%	CD/%	FDTI/%	RV/%
Spring	44.67	42.74	6.53	6.04	0.02
Summer	15.30	42.75	16.00	25.80	0.15
Autumn	4.53	15.03	61.71	18.70	0.03
Winter	36.10	51.70	3.77	8.42	0.01

**Table 5:** Factor detection q-value table from 2003 to 2020.

Variable	2003 Year				2010 Year				2020 Year			
	Spring	Summer	Autumn	Winter	Spring	Summer	Autumn	Winter	Spring	Summer	Autumn	Winter
PD	0.202 *	0.194 *	0.243 *	0.177 *	0.234 *	0.203 *	0.220 *	0.163 *	0.202 *	0.341 *	0.223 *	0.190 *
AREA_MN	0.202 *	0.192 *	0.251 *	0.206 *	0.241 *	0.199 *	0.230 *	0.199 *	0.183 *	0.310 *	0.238 *	0.225 *
AI	0.197 *	0.194 *	0.241 *	0.170 *	0.227 *	0.195 *	0.215 *	0.156 *	0.201 *	0.324 *	0.219 *	0.188 *
P	0.630 *	0.842 *	0.490 *	0.648 *	0.663 *	0.768 *	0.496 *	0.739 *	0.333 *	0.815 *	0.469 *	0.589 *
Air temperature	0.818 *	0.873 *	0.710 *	0.841 *	0.647 *	0.832 *	0.757 *	0.820 *	0.846 *	0.801 *	0.770 *	0.867 *
NDVI	0.525 *	0.544 *	0.634 *	0.573 *	0.563 *	0.525 *	0.450 *	0.629 *	0.451 *	0.458 *	0.468 *	0.649 *
Slope	0.202 *	0.217 *	0.191 *	0.141 *	0.156 *	0.205 *	0.173 *	0.142 *	0.256 *	0.202 *	0.217 *	0.158 *
Aspect	0.022 *	0.027 *	0.027 *	0.052 *	0.034 *	0.025 *	0.018 *	0.045 *	0.025 *	0.038 *	0.023 *	0.052 *
NTL	0.044 *	0.020 *	0.088 *	0.378 *	0.127 *	0.059 *	0.084 *	0.222 *	0.006 *	0.012 *	0.008 *	0.330 *

* Indicates *p* < 0.01.

**Table 6:** Model regression results for summer 2020.

Index	RSS	LIK	AIC	R^2^	Adjust R^2^
OLS	450.859	−1337.611	2697.222	0.882	0.882
MGWR	73.335	2139.357	−2961.946	0.981	0.978

## Data Availability

Data are contained within the article.
